# BeSafe B2.0 Smart Multisensory Platform for Safety in Workplaces

**DOI:** 10.3390/s21103372

**Published:** 2021-05-12

**Authors:** Sergio Márquez-Sánchez, Israel Campero-Jurado, Daniel Robles-Camarillo, Sara Rodríguez, Juan M. Corchado-Rodríguez

**Affiliations:** 1 BISITE Research Group, University of Salamanca, Calle Espejo s/n. Edificio Multiusos I+D+i, 37007 Salamanca, Spain; srg@usal.es (S.R.); corchado@usal.es (J.M.C.-R.); 2 Air Institute, IoT Digital Innovation Hub (Spain), 37188 Salamanca, Spain; 3 Department of Mathematics and Computer Science, Eindhoven University of Technology, 5600MB Eindhoven, The Netherlands; i.campero.jurado@tue.nl; 4 Graduate School in Information Technology and Communications Research Department, Universidad Politécnica de Pachuca, Zempoala Hidalgo 43830, Mexico; danielrc@upp.edu.mx; 5 Department of Electronics, Information and Communication, Faculty of Engineering, Osaka Institute of Technology, Osaka 535-8585, Japan; 6 Faculty of Creative Technology & Heritage, Universiti Malaysia Kelantan, Locked Bag 01, Bachok, Kota Bharu 16300, Kelantan, Malaysia

**Keywords:** AIoT, Gaussian mixture model, smart bracelet, anomaly detection, artificial intelligence, smart PPE, machine learning, deeptech, human activity classification

## Abstract

Wearable technologies are becoming a profitable means of monitoring a person’s health state, such as heart rate and physical activity. The use of the smartwatch is becoming consolidated, not only as a novelty but also as a very useful tool for daily use. In addition, other devices, such as helmets or belts, are beneficial for monitoring workers and the early detection of any anomaly. They can provide valuable information, especially in work environments, where they help reduce the rate of accidents and occupational diseases, which makes them powerful Personal Protective Equipment (PPE). The constant monitoring of the worker’s health can be done in real-time, through temperature, falls, noise, impacts, or heart rate meters, activating an audible and vibrating alarm when an anomaly is detected. The gathered information is transmitted to a server in charge of collecting and processing it. In the first place, this paper provides an exhaustive review of the state of the art on works related to electronics for human activity behavior. After that, a smart multisensory bracelet, combined with other devices, developed a control platform that can improve operators’ security in the working environment. Artificial Intelligence and the Internet of Things (AIoT) bring together the information to improve safety on construction sites, power stations, power lines, etc. Real-time and historic data is used to monitor operators’ health and a hybrid system between Gaussian Mixture Model and Human Activity Classification. That is, our contribution is also founded on the use of two machine learning models, one based on unsupervised learning and the other one supervised. Where the GMM gave us a performance of 80%, 85%, 70%, and 80% for the 4 classes classified in real time, the LSTM obtained a result under the confusion matrix of 0.769, 0.892, and 0.921 for the carrying-displacing, falls, and walking-standing activities, respectively. This information was sent in real time through the platform that has been used to analyze and process the data in an alarm system.

## 1. Introduction and Motivation

There is growing an amount of accessories and safety equipment designated to improve environmental conditions for workers in different sectors. Many companies have increased their investment in developing such settings, especially those whose employees must perform strenuous or risky activities [1,2].

People who work in areas, such as mining or industrial locations, are exposed to different conditions which may have a detrimental impact on their health. For example, there may be a high risk of suffering traumatic injuries, a problem that has always been given considerable importance, given that this type of injuries may be fatal.

The innovation of this study lies in promoting safety in the workplace, by creating an intelligent environment in which the use of data, artificial intelligence, and algorithms, makes the anticipation of emerging risks possible and provides assistance in anomalous situations. Our contributions include:

1. The development of wearable hardware for receiving different vital and environmental signals from specific workers or users.
2. A hybrid integration; a model based on anomaly detection and time series analysis.
3. The connection of artificial intelligence models and wearable devices to a platform for data reception and emission of alerts.

Regarding Machine Learning models, our objective was to present a combination of models on the basis of supervised and unsupervised learning in order to decrease the classification noise, since we trained a model (which is explained later on) of time series with all the data from the wearable device; however, due to the nature of the information, our performance in evaluation was little more than 60%, which is why we decided to split the present work.

BeSafe B2.0 also includes the integration of advanced electronics in textile, this technology is sometimes referred to as “electronic textiles” that causes the fabric to acquire greater functionality; through electronics, the system can capture and send the information obtained from monitoring; moreover, the user can interact with the material. The integration of electronics in the fabric favors its use because it can be worn comfortably, as a bracelet, and does not interfere with the everyday work of the user or employee.

The developed deeptech product is an AIoT (Artificial Intelligent IoT) platform that combines Artificial Intelligence and Internet of Things strategies with real-time data acquisition to generate valuable knowledge. In the first version of BeSafe [3], a platform based on rules through decision trees had been presented. However, no relationships displaying linear behavior had been found; for this reason, this research focuses on the analysis of the datasets acquired by the sensors in the bracelet, implementing human activity recognition combined with other unsupervised learning algorithms to detect anomalies. This allows us to take advantage of the concept of AIoT and offer innovative solutions to the user. The devices integrate a wide variety of sensors that ensure the user’s safety on a daily basis. These sensors measure parameters that imply a risk or an anomaly for the user. Regarding the electronics, different technologies are integrated to enable the user to interact with the platform and monitor their parameters, as well as measure the conditions in the surrounding environment. Currently, this bracelet can be produced on demand, and its development can be adapted to the needs of the target user.

The latest data (from 2019), provided by the Spanish national institute of social security statistics, showed that the number of occupational accidents with sick leave was 650,602 out of a total of 19.75 million workers, while the number of accidents without sick leave was 724,321. Among the accidents with sick leave, 3542 were serious incidents, and 489 were fatal accidents [4]. Depending on the work environment, there are greater or lesser risks which must be carefully analyzed [3,5,6]. Some professions involve the performance of high-risk tasks, where there is a possibility of lethal accidents occurring. However, other factors that trigger discomfort among workers: vibration [7], exposure to the sun, temperature, gas or noise, also introduce risk in the work environment. Other factors that make workers vulnerable include work-related or third party liability. There are occupational risks associated with fatigue and stress, and this is certainly of concern.

The state-of-the art platforms focus on increasing productivity, but few on monitoring the environment or the worker. Moreover, these are designed for either very specific or very general cases, and do not adapt well to the needs of industries. In this regard, our platform meets the needs of connected industry by making it possible to integrate different individual protection devices, which allows for real-time visualization and for the early detection of any anomaly. Currently, data collection has become a requirement for industries as it enables the reduction of times and costs [8,9]. Thus, ensuring the integration, security and quality of the data coming from very heterogeneous sources is essential [10,11]. The platform incorporates fusion algorithms and data mining processes of the different connected devices and a knowledge base to reduce the impact of any anomaly, comprising a Smart Data system capable of providing intelligent responses [10]. It uses a methodology composed of algorithms adapted to the obtained information, offering a knowledge base for predicting anomalies that provide great value [12]. The platform incorporates the use of neural networks, fuzzy logic, Bayesian networks, decision trees, and other hybrid inference and artificial intelligence techniques [13], which extract key infromation from large volumes of data. The platform incorporates virtual organizations responsible for the fusion of information from the data sources of multiple domains. When linked together, they further facilitate the performance of a complete analysis.

Human activity recognition (HAR) has attracted the attention of the scientific community because it provides personalized support in different applications since the 1980s, and it has been studied extensively in the last decade. Depending on the purpose of the application, contiguous portions of data streams are used to detect and classify human behavior, preprocessing them transforms the raw signal data into feature vectors. The range of cell phone applications is massive [14], especially in health monitoring, which can be associated with a context and tracked. Some of the most notable examples are detecting movement, when a person is walking or running [15,16,17], tracking emotional state [18], or classifying patterns in sleep and exercise [19]. In this regard, BMI160 IMU has been chosen for data collection, while Gaussian Mixtures, through long-term memory, have been used as a model for the classification of human activity.

Thus, it is extremely important to provide increasingly robust devices capable of identifying anomalies on the basis of the information read by specialized sensors. All this can be achieved through Information and Communication Technologies, together with Artificial Intelligence and electronics.

This research proposes the detection of anomalies through a multisensorial intelligent bracelet so that information can be collected and transmitted for decision-making. This proposal has been implemented in a real environment, as detailed further in this work. As it is a wearable device, the user’s behavior can be analyzed. Among its capabilities, it can measure parameters, such as pulse and temperature, and detect falls or accidents. It sends alerts whenever an anomaly is detected in the environment, thanks to an intelligent platform that provides total support to the operator and other workers.

The remainder of this work is organized as follows: Section 2 describes an overview of the related literature. Section 3 details the system design. In Section 4, we present the electronic system based on a multisensor bracelet where we apply short term memory (LSTM) through the Gaussian mixture model (GMM) and the transmission of information. Thanks to these, it is possible to detect anomalies with the use of a dataset and the implementation of a model for the classification of human activity. Finally, in the last section, we present some conclusive remarks and future lines of research.

## 2. State of the Art

Over the last decade, platforms implementing predictive maintenance strategies have been introduced on the market [20,21,22,23,24]. These solutions monitor machines through the capture of data by sensors, making it possible to maximize the availability of the machines involved in industrial processes, increasing the efficiency of the maintenance process. The emergence of technologies, such as BigData, has also favored the processing of real-time data, to which data mining techniques can be applied in large-scale industrial processes [25]. Another current trend in this type of platforms is to use cloud architectures to process large amounts of data, offering solutions that are framed in software, platform and infrastructure. This type of architecture, combined with virtual organizations of agents, allows the platform to be modular and scalable, facilitating the incorporation of new vertical solutions integrated in the platform’s horizontal design [26,27]. In addition, with the use of technologies, such as edge computing, it is possible to propose environments capable of processing information close to the device, which makes it possible to manage data more efficiently and rationally [28], many of these platforms are based on models with rule structures, where depending on certain conditions decisions can be made about the information being processed in the cloud. However, our proposal is extended by integrating two artificial intelligence models that work together to mitigate false alarm errors. The use of the different enabling technologies of Industry 4.0 has led to the creation of new platforms and to the improvement in the quality of the obtained information. Industry 4.0 platforms are able to process data and understand their meaning; a feature that can revolutionize today’s industries, as the majority of current platforms do not apply these new technologies [29,30,31,32]. Recently, relevant research studies have been conducted in the area of smartwatch wearables, for instance, to prevent the spread of COVID-19, as well as to identify the disease at a much earlier stage, reduce death rate, and monitor the user’s body temperature, heart rate, and blood pressure [33]. Other studies propose other smartwatch functionalities, apart from parameter measurement. For example, the research of Adjiski Vancho et al. involved the development of an architecture that can be used in underground mining and that uses sensors attached to regular PPE clothing, including hard hats and safety glasses. These sensors are connected to the smartphone and smartwatch via Bluetooth low energy sensors, to provide real-time safety, situational awareness, and predict health and safety incidents before they occur [34].

**Figure 1:**
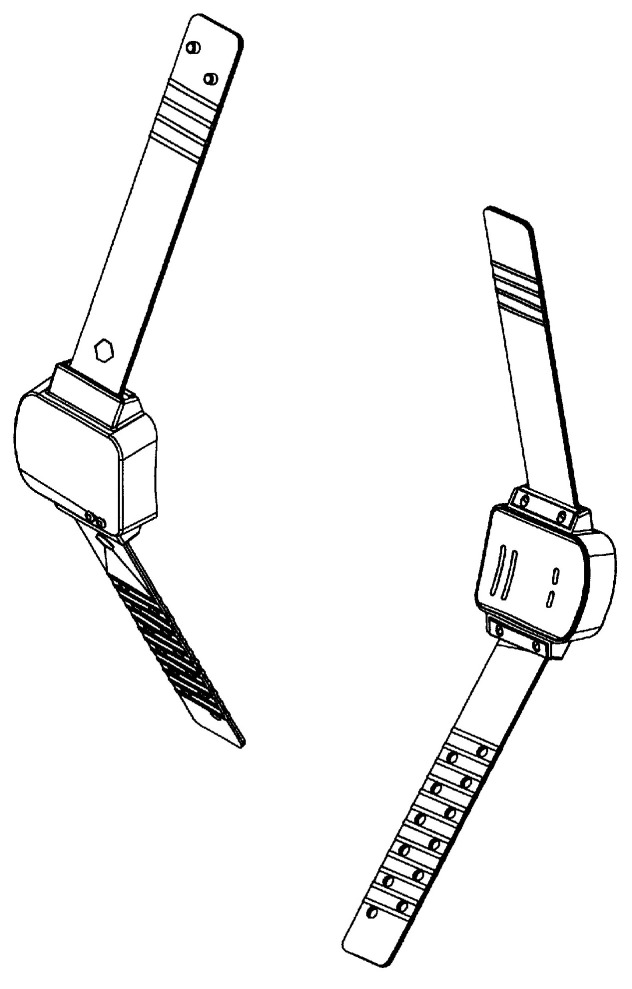
Patent watch/bracelet form USD535205S1 [35].

Regarding the safety of workers and creating a safe working environment, wearable devices have captured the attention of industries and the academic community. This fact is evidenced by the growing tendency of organizations towards the research and development of these products, due to the multiple fields in which they can be useful: sports, security, health, entertainment, etc. There have been several advances and proposals in the field of electronic systems, which focused on the ability to monitor users’s vital signs. Among those proposals is patent USD535205S1 [35], made in the United States and invented by Walter H. Frederick et al., with a clock design, as shown in Figure 1.

Another patent registered in the United States was the device US20170027511A1 invented by Robert A. Connor [36]. The patent is currently active, this gadget is a device that can be carried on the arm with adjusted bio-metric sensors. These can be spectroscopic sensors that project light onto the surface of the arm at different angles. Alternatively, these sensors can be electromagnetic energy sensors that measure the impedance, resistance, conductivity or permittivity of tissues. One of the main approaches of this patent is to measure the levels of oxygen, glucose, hydration, and even the heart rate of the individual; that is, this patent focuses on the vital signs of the person, where the proposed design is included in a general way. Our aim is to focus on a tool for the environment that workers are in, i.e., analyzing changes in user behavior to detect problems, such as falls, as this has always been a problem in industrial companies.

Finally, the U.S. patent, US20190064792A1, invented by Charles Howard Cella et al., is a monitoring and surveillance system for implementation in a data collection environment. It operates through the Internet of Industrial Things (IIoT) with intelligent data management for industrial processes, including an analog sensor. A data collector can be added to this system and coupled with multiple analog sensors. Likewise, the data storage and data analysis circuit’s structure enables it to analyze the collected data and select a plan from among numerous data collection management plans, to later analyze the received data and decision management for actuators. This work is quite similar to ours; however, we focus on real-time analysis for immediate decision-making, as our goal is to reduce response time when there has been a fallout from industrial staff or the environmental issues that compromises workers’ health.

In September 2016, Michele Magno et al. [37] presented the design and implementation of a sensor-based, energy-collecting smart bracelet called InfiniTime, which also underwent field evaluation. The system has a wearable design, and it integrates temperature sensors and an accelerometer, as well as an ultra-low power camera and a microphone. The purpose of the bracelet is self-sustainability by using solar cells with modest levels of indoor light and thermoelectric generators (TEG) with body heat temperature gradients.

In November 2013, Martin Ouwerkerk et al. [38] developed a wireless sensor bracelet, its design based on the knowledge gained from a predecessor sensor bracelet, and the authors gave reasons for the sensors they had chosen. The disadvantage they present is the battery performance of the product software, which did not last 7 working days; therefore, they addressed it through software optimization, which meets the design objective of battery life. In addition, in March 2015, Giancarlo Fortino et al. [39] proposed *C-SPINE*, a framework for collaborative social security networks (CBSN), where collaboration is based on interaction and synchronization between CBSNs and collaborative distributed computing over collaborating CBSNs. CBSNs are body sensor networks (BSNs) that have the ability to interact and support each other to perform a task. To demonstrate their effectiveness, they implemented e-Shake, a CBSN collaborative system for detecting emotions. This system must acquire multisensor data to perform automatic detection. Regarding the power consumption of our device, we have adapted it to be as low as possible (which will be explained, together with the other hardware details, in Section 3).

In 2017, Singh Ericet et al. [40], addressed graphene-based materials and their possible applications in flexible and extendable wearable electronic devices, where graphene is one of the nanomaterials with a broad variety of uses. In addition, it has other implicit properties that make it suitable for use in fields, such as cancer detection or chemical detection. Moreover, the authors discussed the role of graphene in the manufacture of flexible gas sensors for the detection of various hazardous gases; this type of sensor could be considered in a future research to improve the current proposal. In November 2015, several transparent and stretchable sensors (TS) with high optical transparency were introduced by Trung Tran Quang et al. [41]. The TS gate sensor array has a high response to temperature changes occurring in objects and the human skin. Since the emergence of this technology, a growing amount of highly compatible devices have been developed to be worn and used by specific users. This work showed the applicability of the TS gate sensor array in skin electronics for the recognition of human activity.

Concerning the parameters that can be measured by this type of device, we should highlight GPS, for the location of the user carrying the Smartwatch [42,43,44,45]. In other cases, triangulation [46] offers a more precise system which is used by dementia patients to improve their living conditions [47]. Research has also been done on measuring heart rate [48], and some studies in literature employ an optical sensor for heart rate monitoring [49,50,51]. Other studies deal with the monitoring of heart frequency, using a technique called photoplethysmography [52,53,54], thanks to which, from a beam of light, the volume of the determined organ can be measured, and, in the majority of cases, it is used to calculate the amount of oxygen in the blood.

Human Activity Recognition (HAR) has become of great interest in several sectors, including PRL. Its development is favored by the development of multisensor systems, which use a combination of inertial measurement units (IMUs) on the body. The aim is to identify and detect activities; however, this is a complex task as the data is ambiguous or noisy. Therefore, Machine Learning-based models are required to learn from the data to extract knowledge and detect and classify behavioral patterns [55,56,57]. Numerous works exploring the use of techniques that focus on Naive Bayes (NB) [58], Decision Trees [59], Hidden Markov Modeling (HMM) [60], Neural Networks (NN) [61], Support Vector Machines (SVM) [62], and Deep Belief Networks (DBN) [63] are some of the most relevant implementations. Another noteworthy application is the use of a three-axis accelerometer, which, helped by an algorithm, can detect the number of steps taken and monitor the user’s physical activity [45,46,47,49] and even detect falls [53], epileptic seizures [43], and degrees of shaking that happen to people with Parkinson’s disease [64]. It is remarkable to know that thanks to the combination of accelerometers, gyroscopes (IMUs) and algorithms, we can reach a high precision [50,65], as demonstrated in the Smartwatch Pebble which used the CUSUM algorithm [66] and advanced sorting and filtering techniques from Kalman for the subsequent detection of falls, using an accelerometer, a gyroscope, and contact sensors to perform an initial evaluation. In addition, to filter out the noise and avoid errors [46], the Moving Average Filter (MAF) was used.

There are intelligent algorithms capable of gathering the data, analyzing it, and deciding whether it is a false positive, an expected value or whether an emergency is occurring, thanks to the prior programming of the data monitored. Minimum and maximum values are programmed, and, if we are in between them, it means that the patient’s condition is normal. It is essential to highlight that this algorithm learns; that is, if the system alerts of an abnormality that is later assessed by the doctor as a false positive, the algorithm would not make that mistake again. When it has accumulated 48 measurements, it sends a report to the doctor about the user’s condition [51]. Another example of data collection and analysis is the Rapid Miner [67] or the use of an expert system, such as KBS, capable of making decisions and taking action in cases where the patient exceeds the established thresholds [48]. In terms of monitoring the psychological state of the user, we find Smart Personal Health Advisor (SPHA) architectures [45] which provide the user with a personalized life guide to their daily activities and interaction with their environment. For monitoring purposes, smart textiles are used to collect the user’s physiological data, with sensors to measure the electrocardiogram, temperature and the amount of oxygen in the blood.

Regarding the noise that can be generated in an environment, in September 2004, an active noise cancellation methodology, using a MEMS accelerometer to recover the signals from the sensors that have been corrupted due to body movement, was proposed by Asada H. Harry et al. [68]. The procedure was proposed for a finger ring photoplethysmogram (PPG) sensor, in which the signal is susceptible to the movement of the wearer’s hand. The purpose was to recover the corrupted PPG signal. The positive or negative relationship between the acceleration and the distorted PPG signal is analyzed, and a low-order Finite Impulse Response (FIR) model is obtained that relates the signal distortion to the hand acceleration.

Regarding artificial intelligence models, in 2009, Reynolds [69] presented the main characteristics of the GMM, which is a parametric probability density function represented as a weighted sum of the densities of the Gaussian components. Other investigations have also been carried out on the same AI model [45,70,71]. This model has been widely used to detect anomalies due to its ideal characteristics [45,72,73,74]; therefore, in the present paper, it will be used to find alterations in the data that have to be in a certain distribution in order to detect possible problems in a work environment where the short- or long-term risk of suffering an accident, disorder, or fracture is high [75,76,77,78,79,80].

Among the wearable products, we find many commercial products that allow to monitor vital signs and enable interaction, such as SmartWatch (Apple Watch Series 5, Xiaomi mi band 4, Amazfit, Huawei Smartwatch, Samsung galaxy watch 2, etc.). Their technological features with a wide variety of sensors and functions make them very attractive for daily use. They can help us in areas, such as health monitoring, by sending notifications on training completion, goal setting, sleep monitoring, heart rate monitoring, all-day heart rate review, resting heart rate, heart rate table, and inactivity alerts. We can also use them in our training or use other features. They offer stopwatches as alarms and phone notifications, incoming calls, message and calendar notifications, application notifications, weather forecast, find the phone, unlock the phone, and event notification. In terms of sensing, they have a GPS, a heart rate sensor, an accelerometer, a gyro, a magnetic sensor, a pressure sensor, an ambient light sensor, a capacitive sensor, NFC, LTE, Wi-Fi, and others [5,6,81,82].

E. C. Nelson et al. (2016) [83] demonstrated, through a survey in which data was collected on the internet, that people can influence their motivation and actions if they commit to the self-observation of their activity. The theory further postulates that the gathered self-diagnostic information has an important self-motivating function and gives people the ability to set goals for objective improvement and gives them the feeling that they can regulate those goals themselves. Thus, the success of self-regulation depends partially on the fidelity, consistency and temporal proximity of self-monitoring [84]. If we bring this result to the work environment, the worker may feel more confident in performing their tasks while wearing the bracelet, which may even improve the quality of their work and health. K. J. Kim et al. (2015) [85] examine several psychological factors closely associated with wearing technology and explain how these factors contribute to determining the user acceptance of smartwatches by integrating them with the technology acceptance model (TAM), one of the most widely used theoretical models for studying end-user acceptance of ICTs. The investigations that propose wrist bands with wearable monitoring and sensor networks are compiled in Table 1.

The novelty of the proposed solution lies in its holistic approach. It uses artificial intelligence models combined with wearable electronics and IoT devices to provide it with greater capabilities than the solutions developed until now, creating a much more favorable environment for the personal protection of workers. Compared to other state-of-the-art developments, the novelty of this proposal is the development of a new wearable device for the measurement of different vital and environmental signals from specific workers or users. In addition to the ability to connect the bracelets to the platform, it is also possible to integrate other devices that provide the system with complementary information. These devices are can be used in different environments, thus achieving a modular design that is valid for the different problems we may encounter in different work environments. Another novelty of this research is the union of artificial intelligence models and wearable devices in a hybrid platform, offering data reception and alarm generation functionalities through the hybrid integration of model-based anomaly detection and time series analysis. Regarding Machine Learning models, our objective was to present an union of models based on supervised and unsupervised learning, in order to decrease the classification noise since we trained a model of time series (which will be explained later) including all the data from the wearable devices; however, due to the nature of the information, our performance in evaluation was little more than 60%, which is why we decided to split the present work.

## 3. The BeSafe Platform

The main objective of this investigation is the presentation of a platform to improve the safety of operators’ in their work environment, and it involves research into information and communication systems equipped with ambient intelligence. Due to previous research, this product is already at stage 2 of development, and is being developed further in the current study. Changes have been made in terms of ergonomics, speed of receiving information and safety, and previous versions have been referenced [81].

To develop this platform, the requirements have been specified, and the architecture has been defined to identify the most common risk situations and generalize them, achieving the greatest possible impact through a limited set of actions. Given the large amount of information on this topic, an analysis has been carried out of the recommendations that appear in the regulations and risk prevention manuals, with the aim of identifying the risks that could be feasibly covered, given their compatibility with the developed device. On this basis, the functionalities to be incorporated in the proposed solution have been defined, as well as the technical aspects of the device implementation, such as the design of an architecture, the definition of the components and the connectors, and the correct identification of the flow of information through the different modules. Using the formalization of the model, the rules for associating the risks with a set of preventive measures have been created, given the context of the situation.

Version 2.0 has been completed after carrying out the evaluation process, resulting in a control and visualization platform in which hybrid artificial intelligence algorithms are integrated for the detection and prevention of risks and accidents. As part of platform development, algorithms have been designed for decision-making and for the identification of risk situations and the identification of these scenarios in the database, so that the worker can be notified.

The last phase sought to integrate and validate the system in a simulated environment by deploying the platform and interconnecting it with the bracelet, helmet and belt, checking that the developed functionalities met the requirements specified in the first phase of the project with the corresponding advantages provided by the use of data analysis, prevention algorithms and the use of the control platform. The functional solution has been validated in different work environments, where its correct performance has been evaluated.

The research process has demanded the application of different agile methodologies that ensured the obtained results would be aligned with the initial objectives. Starting with the collection, organization, and the subsequent analysis of the information, so a time frame is set for the achievement of intermediate stages, and a metric system is established to evaluate the results.

### 3.1. BeSafe B2.0 Platform Design

The prototype that is being sought is a bracelet with a wide variety of sensors that increase the users’ safety in daily life. Its key objective is to detect anomalies or any situation that may put the user at risk. To this end, a review has been carried out of the different technologies available on the market in the field of interaction and monitoring of both the user and the environment as shown in Table 2.

**Figure 2:**
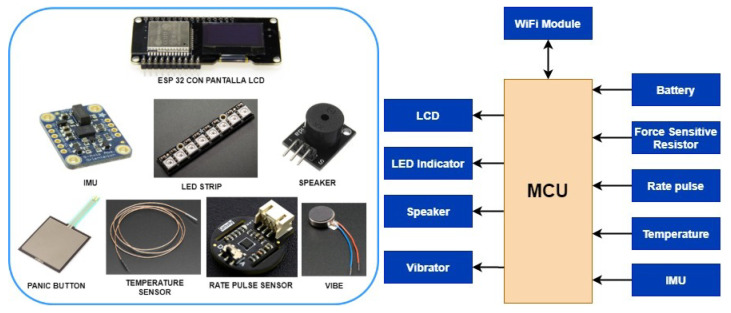
Electronic modules chosen.

Below are all the technologies that have been chosen for implementation, as shown in Figure 2 and Table 3.

It has been decided to use an optical sensor to measure heart frequency, after analyzing the different types of sensors available in this field, such as those based on photoplethysmography techniques, which are much more precise and capable of providing us with important information, for example, the amount of oxygen in the blood. However, this technique performs the analysis on the fingertip and not the wrist, since our proposal is a bracelet, we discarded this option, and an optical sensor has been chosen.

The usual optical sensors are made up of photodiodes, i.e., sensors that emit light. They work as follows: We have two light emitters and two light receivers. Once the sensor is activated, the light emits a return reflection that indicates to the bracelet the level of blood in that particular place. Our heart pumps blood causing the reflected light to vary at each instant, this makes it possible to estimate the measurement of the pulses per minute. An example of this type of sensors would be the pulsometer, which can electronically control the heart rate through the electrodes in contact with the skin. Another example is a fiber optic sensor in which the pulses modify the shape of the fiber optic, which in turn causes a variation in the reflection of the light that circulates through it.

Measuring the user’s body temperature through an infrared sensor may be slightly imprecise since the device is worn on the wrist and may lose contact with the skin. For this reason, we select a Thermocouple Type-K Glass Braid Insulated Stainless Steel Tip from Adafruit (New York, NY, USA), which are best used for measuring surface temperatures. Because thermocouples are very sensitive, we have included a MAX31855 amplifier from Adafruit (New York, NY, USA), with a cold-compensation reference.

**Figure 3:**
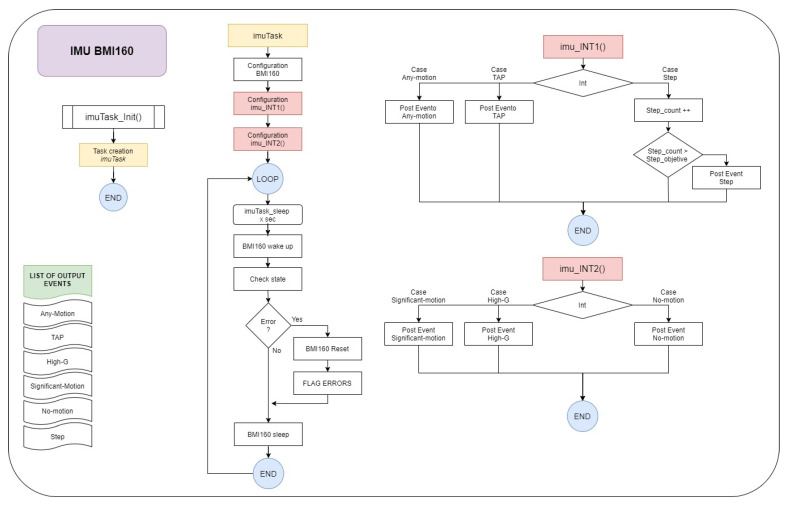
Electronic modules chosen.

For human activity recognition (HAR), a BMI160 inertial sensor (IMU) from Bosch (Stuttgart, Germany) has been selected for I2C communication without an external magnetometer. The BMI160 is a highly integrated, low power inertial measurement unit (IMU) that provides precise acceleration and angular rate (gyroscopic) measurement. The BMI160 integrates: 16-bit digital, triaxial accelerometer and 16-bit digital, triaxial gyroscope. It is connected to the ESP32 via an I2C port. As a relevant feature, it incorporates two interrupt lines INT1_ACC and INT2_ACC, managed by a specific program that configures them, shown in Figure 3. Through Imu_INT1 the Any-motion, Tap and Step interrupts are detected, and, with Imu_INT2, the Significant-motion, High-G, and No-motion interrupt are detected. At each interruption, the relevant event is posted, thus facilitating the classification of the activity and interpretation of the different types of movement.

The panic button is used by the user to warn of any anomaly or a risk they are experiencing. To do this, we have contemplated the use of piezoresistive sensors that, by means of a certain pressure, can activate an alarm or, on the contrary, can cancel a false alarm.

In addition, it is worthy of mention that the information collected by the bracelet is sent to a control and interactivity interface. The use of adapted control panels and interactive devices in the industrial environment is becoming increasingly common, partly due to the need to manage large volumes of data that are beyond the reasoning capacity of human beings. In this respect, a system has been developed that allows information to be conceptualized and displayed understandably, for rapid and intuitive assimilation. This solution makes it possible to improve users’ reaction time and eliminate any doubts that may arise in a critical situation. In the face of a situation that creates stress and uncertainty, it is always more advisable to have a guide that provides simple and effective recommendations.

**Figure 4:**
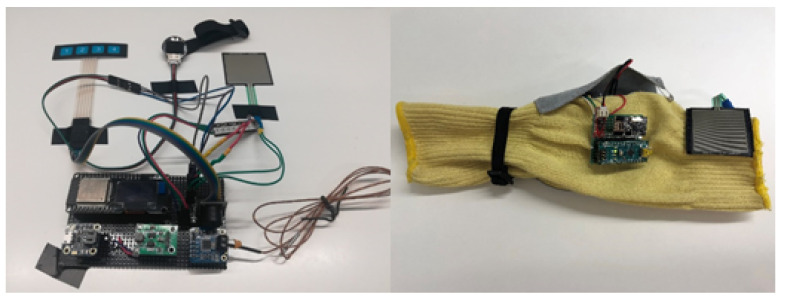
Bracelet with electronic components and their deployment on the fabric.

Figure 4 below shows the board with all the connected sensors and electronic components. Note that the external elements (image on the left) are integrated in the bracelet (image on the right).

**Figure 5:**
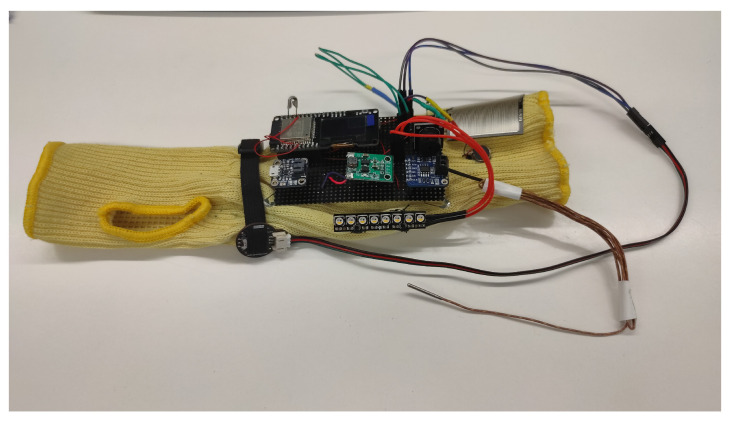
Bracelet with the electronics and sensors included.

The bracelet electronics consist in electronic components welded to a flexible protoboard. These are ESP32 with LCD display, Buzzer and monitoring sensor drivers. On the one hand, the electronics responsible for monitoring vital signs and detecting anomalies are attached to the textile part of the bracelet, as shown in the image on the right in Figure 5, including the temperature sensor, pulse sensor and panic button. On the other hand, we have the possibility of visualizing the alarms by means of a LED strip that is also on the outside. The power supply is provided by a 3.7 V Lipo battery, connected to the power module, which charges the bracelet.

The complete system may be made up of multiple modules that take different measurements of the worker’s condition and/or their environment. Each bracelet has a communication channel that is used to send the measurements obtained to the concentrator node.

The concentrator node consists of a Raspberry Pi, model 3B, which mounts Ubuntu Mate as its operating system. This node aims to collect the measurements sent by the different devices, store them and route them to a display mechanism. To allow for communication between the measurement devices and the concentrator, the latter provides the system with a Mosquitto server, which implements the MQTT communication protocol. The MQTT protocol has been chosen because it is adapted to the volume of data that needs to be transmitted and also provides native security standards, such as TSL (Transport Layer Security) and SSL (Secure Sockets Layer). The data is sent between the ESP32 microcontrollers and the concentrator in JSON format. In previous work, NVIDIA Jetson Nano has been used, which enables in-hardware processing and edge computing [82].

**Figure 6:**
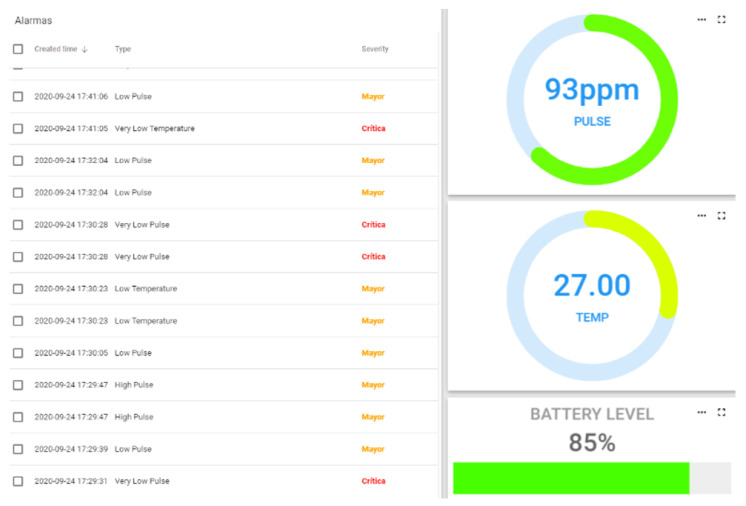
BeSafe B2.0 Platform, bracelet alarm panel.

The data received on the concentrator is collected by software developed in Python 3. This software is responsible for carrying out the storage tasks and sending the data to the display dashboard. For storage, a database implemented in “MongoDB” is used, where the data received by the MQTT protocol are stored. MongoDB has been used because it is a document-oriented database, which provides the possibility of storing the JSON files received without previous transformations. As a dashboard tool, Thingsboard has been chosen, and it provides a versatile and user-friendly data visualization system, as shown in Figure 6. It has a rules engine that allows it to respond to events and record critical situations. The alert system is implemented on this rules engine. The MQTT protocol and the JSON format are again used to send the data between the hub and the Thingsboard servers, both local and remote. In this case, Thingsboard provides its own MQTT server, so it is not necessary to use Mosquitto.

**Figure 7:**
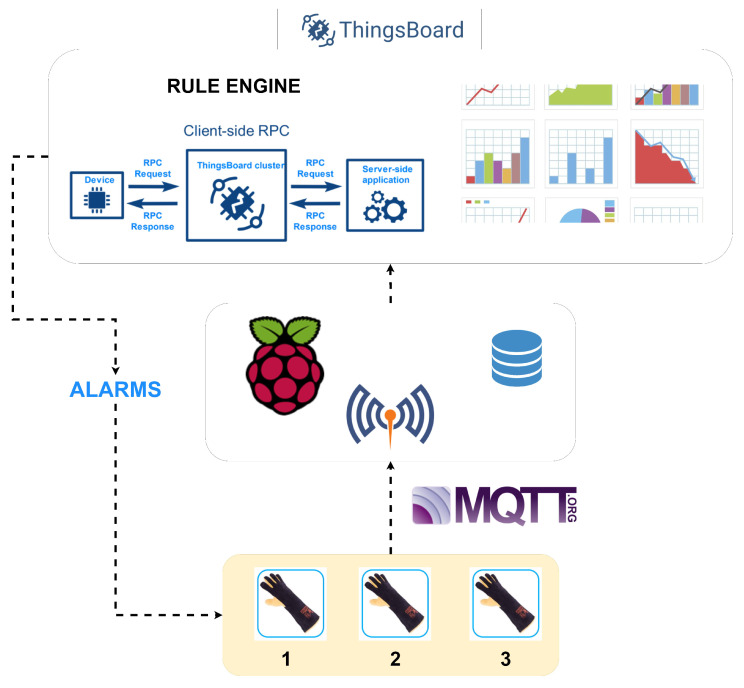
System architecture.

The data stored in MongoDB is processed by the software developed in Python 3, on a remote machine, more powerful than a Raspberry Pi. The SKLearn library (Scikit-learn) from Python is used to process this data. This library is oriented to processing large blocks of data, including classification algorithms, regression and group analysis. Through the analysis of these data, it is intended to find behavior patterns that allow to predict anomalous situations. The system architecture is shown in Figure 7 below.

**Figure 8:**
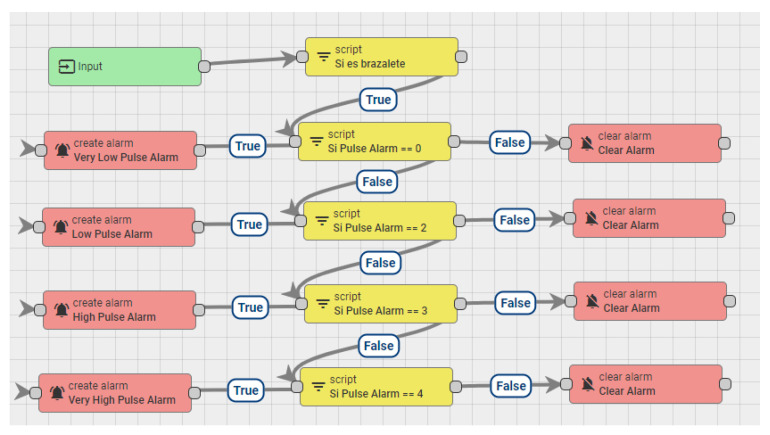
Bracelet pulse alarm configuration.

The operation of the bracelet is based on the information it collects from the components: temperature sensor, IMU, cardiac pulse module and the resistive force sensor. It also analyzes the percentage of battery charge. The temperature sensor is used to take body temperature readings, normal body temperature is between 35° and 38°, which is an essential variable when analyzing the worker’s medical condition. If the temperature is lower or higher than the established threshold, an alarm is set off to warn the worker, as shown in Figure 8.

**Figure 9:**
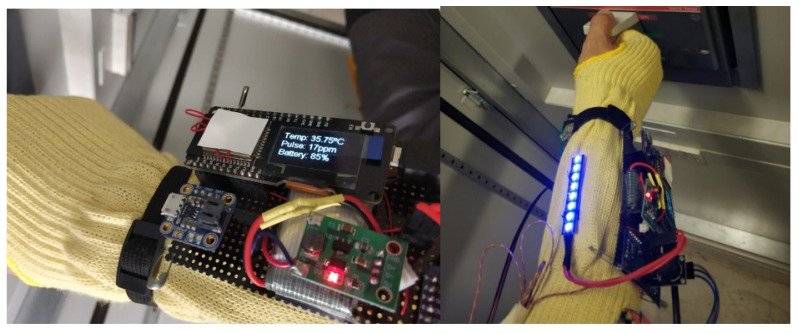
Picture of the data displayed on the screen and of the active pulse alarm.

The cardiac pulse sensor is responsible for analyzing the worker’s heartbeat per minute (bpm). The normal heart rate in the human body is between 50 and 110 bpm, if any of the mentioned activation thresholds are exceeded, a notification is issued in order to take action in response to the situation. The force-resistive sensor is used to activate the panic alarm or to cancel a false alarm. Figure 9 shows the operation of the bracelet and the data on an LCD display.

### 3.2. Data Analysis and Detection Proposal

**Figure 10:**
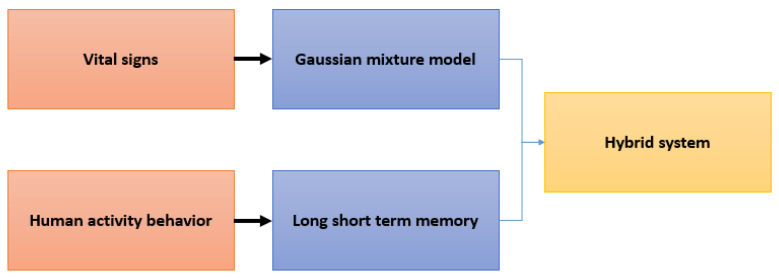
Data analysis through the union of a model based on anomalies and the following one based on time series.

The Gaussian Mixture Model is a tool that allows us to group information depending on its characteristics, one of its many advantages is the ability to choose components automatically. This section explains how the information has been analyzed and the different segmentations that have been made of the dataset. The inputs of the model are the user’s body temperature, heart rate and a variable indicating the state of the bracelet (the optimal state is determined by the battery and the physical coupling, where the output is the type of distribution to which it belongs). Considering that this is unsupervised learning, depending on the number of components involved, there will be some associated labels. In this study, we used Fisher’s analysis to find the populations and on its basis, and the number of components has been defined to be 4. It can expected that a label among these 4 situations categorized in a real working environment as a heart attack alert through agitation or falls, problems in the user’s body temperature, and, in these situations, the virus called COVID-19, is also quite feasible to detect users at risk. Moreover, LSTM is used to analyze the data coming from the IMU of the electronic system. These data are analyzed separately to reduce the noise that can originate from the combination of both and, thus, generate a system with a hybrid analysis, in time series and anomalies; see Figure 10.

Due to the use of these models in a concise manner, the state of the art has shown that LSTMs work extremely well for time series problems, even if there is no linear correlation, and GMMs have also been used feasibly in alarm systems.

#### 3.2.1. LSTM

As it is well known, there are models proposed specifically for time series analysis; in our case, we make use of one of the most used in the literature for its robustness, the LSTM, a model focused on information recall. This model is based on recurrent neural networks; see Reference [86] to find the details of the theory. In the area of Human Activity Behavior, different situations are studied to try to find a relationship over time with the objective of modeling different circumstances. Our work is focused on the industrial field; therefore, talking to experts in this area, as well as performing a quantitative analysis of the most common activities, they were found to be carrying-sliding objects, walking-standing, and our objective is clearly to analyze shocks.

**Figure 11:**
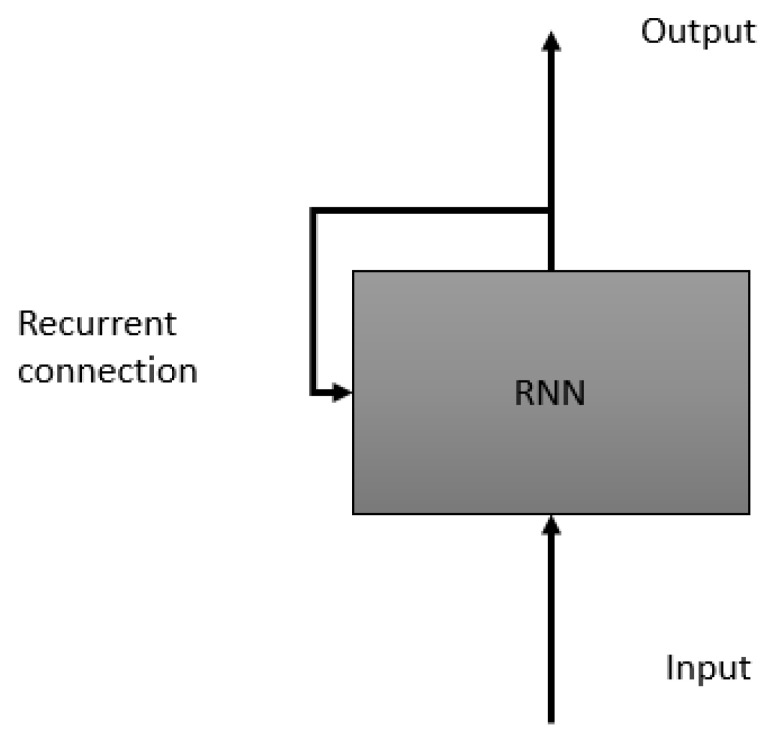
Concept of Recurrent Neural Networks.

As it is well known [87], an LSTM is a variant of a Recurrent Neural Network, where the basis is that a sequence can be processed by iterating and maintaining a certain nature containing information in the past. Figure 11 shows the concept behind this. The RNN has the particularity of eliminating or forgetting the information between the processing of two independent sequences, which is why it allows finding patterns on specific time series. Our dataset was taken at a frequency of 50 Hz for 4 consecutive hours from 5 different employees, where the accelerometer data provided by their smartphones was acquired simulating performing the 3 mentioned activities. It is worth mentioning that, since we have an imbalance of data, we performed an oversampling on the falls in order to have a smaller bias.

**Figure 12:**
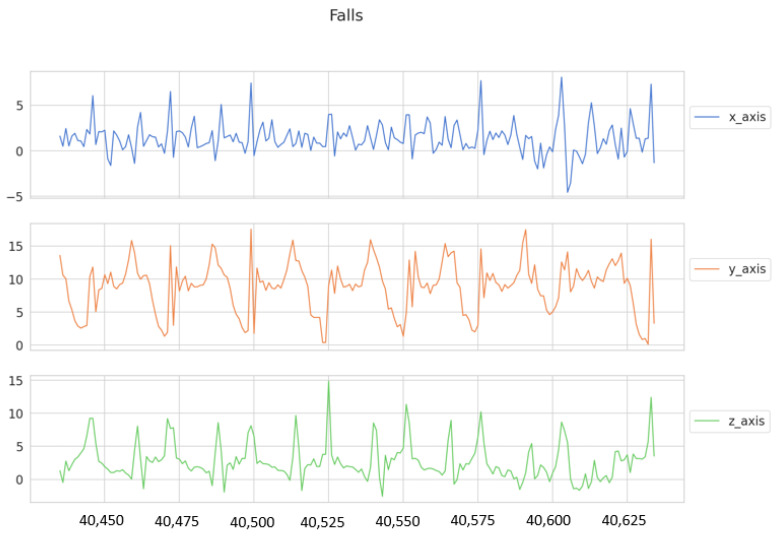
Behavior of the time series for the fall tag.

One of the ways in which we can generate the hypothesis that our data will be modeled adequately is through finding visual patterns; see Figure 12, where we have displayed the information concerning the drops for the 3 axes, and we can clearly see that there is a similar behavior.

Thus, our objective is to detect possible risks, mainly falls, in order to improve the response time to a dangerous situation. Shocks are not a well sample that can be easily acquired; therefore, several people from the industrial area were asked to simulate this action. Despite the efforts, only a time series of 10,000 sample size was obtained for the frequency being used (50 Hz as mentioned above). Therefore, data augmentation was used. Data augmentation works through random transformations, depending on the data you want to use, such as images, raw data, or, in this case, time series, and the transformation may vary.

For the augmentation of the time series data, we rely on the following reference [88]: Suppose we have the time-series $\left\{A_{i}\right\}$, with 1≤i≤n. And suppose also that we have ϵ that meets the following condition $0<\epsilon <\left|A_{i+1}-A_{i}\right|\forall i\in \left\{1,\cdots ,n\right\}$.

We can construct a new time series $\left\{B_{i}=A_{i}+r_{i}\right\}$, where $r_{i}$ is a realization of the distribution $N(0,\frac{\epsilon }{2})$.

Then, instead of minimizing the loss function only over $\left\{A_{i}\right\}$, we do that also over $\left\{B_{i}\right\}$. So, if the optimization process takes *m* steps, we have to *initialize* the predictor 2m times, and we will compute approximately 2m(n−1) predictor internal states. Thanks to the increase in time series data, we went from 10,000 samples for the fall class to more than 70,000 samples for the fall class.

**Figure 13:**
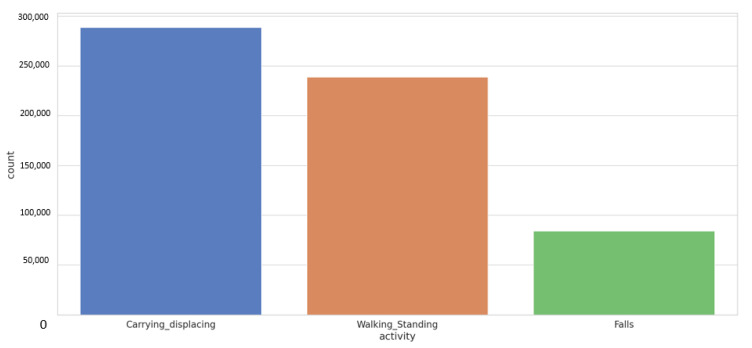
Histogram diagram of number of samples by class.

The dataset was composed as in the following, Figure 13, and it should be considered that the dataset for the different actions obtained constituted an unbalanced set, to which can be added the different performance shown below in the confusion matrix. The first two activities carrying-displaying and Walking Standing were acquired during normal performance. However, for the Falls class, the simulation had to be performed by different workers on different surfaces, as can be seen, and it was the class with the smallest number of data.

**Figure 14:**
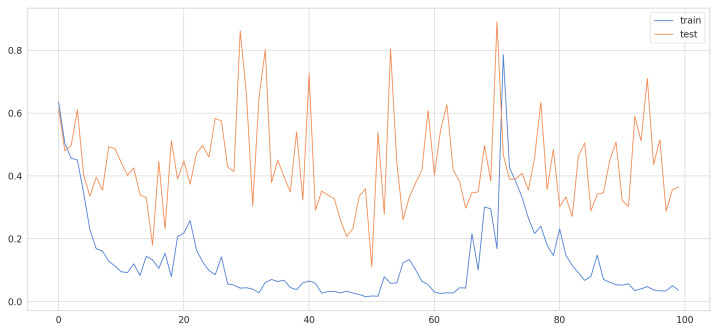
LSTM history loss training and testing dataset.

Now that we know the input for our model we proceed to train the LSTM, of which we have the following characteristics: we have 200 time steps, with a training set dimension $\in R^{3}$ (12,670, 200, 3), it is a sequential model, where we have 128 units for the LSTM, our learning rate 0.5, a hidden layer with activation function relu and of course output layer with softmax it is already a multiclass classification problem. We train it with 100 epochs where, as shown in Figure 14, you can see the history of the loss in the training and testing set.

**Figure 15:**
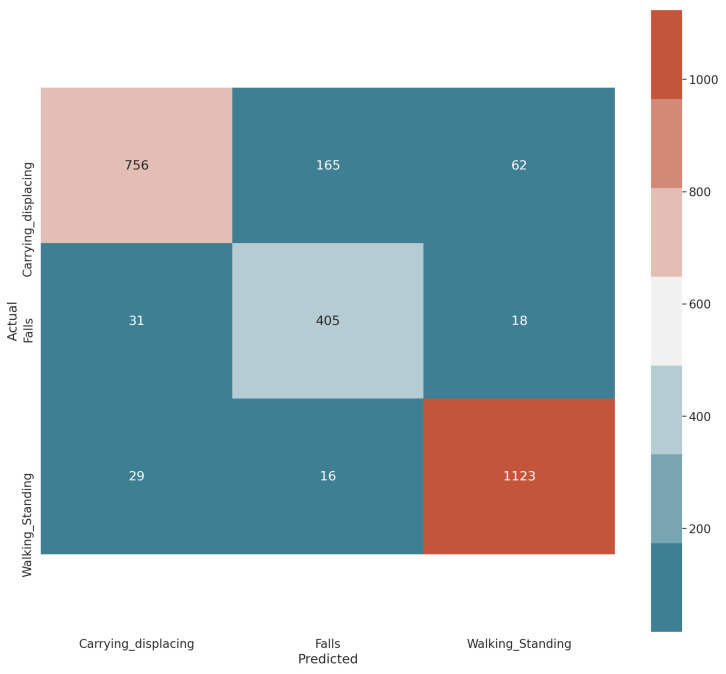
Confusion matrix LSTM.

To come to the result of the confusion matrix, a combination of hyperparameters was performed where the number of units was varied between 64 and 128 for the LSTM, as well as the learning step between 0 and 0.5, with steps of 0.05 and number of epochs between 10 and 100, where, as in Figure 15, it is equivalent to the output given the configuration described above. We will now proceed to explain the anomalies part.

#### 3.2.2. GMM

On the basis of the state of the art and the model proposed to solve the problem, the mathematical basis of the GMM. As mentioned, the GMM is a parametric probability density function represented as a Gaussian weighted sum of the component densities. In GMM, parameters are estimated from training data using the iterative Expectation-Maximization (EM) algorithm or the Maximum A Posteriori Estimation (MAP).

Unlike *K-Means*, this method’s purpose is to fit Gaussian *M* to the data. After this step, the Gaussian distribution parameters, such as mean and variance for each cluster and the weight of a cluster, are found. Finally, for each data point, the probabilities of belonging to each of the clusters are calculated. A model based on a Gaussian distribution can be written as seen in Equation (1) [89].

(1) $$p\left(x|\lambda \right)=\sum_{i=1}^{M}w_{i}g\left(x|\mu_{i},\sigma_{i}\right),$$

where Equation (1) establishes a univariate case; in other words, it is a one-dimensional model. The definition continues in Equation (2).

(2) $$g\left(x|\mu_{i},\sigma_{i}\right)=\frac{1}{\sigma_{i}\sqrt{2\pi }}exp\left(-\frac{{\left(x-\mu_{i}\right)}^{2}}{2\sigma_{i}^{2}}\right),$$

where, for both Equations (1) and (2), μM= mean and σM= variance for each of the *M*-th element, *x* is a *D*-dimensional continuous-valued data vector (i.e., measurement or features), and $w_{i},i=1,\cdots ,M$ are the mixture weights.

When the case being addressed has multivariate problems Equation (3) is applied.

(3) $$p\left(\vec{x}\right)=\sum_{i=1}^{M}w_{i}g\left(\vec{x}|\vec{\mu_{i}},\sum_{i}\right),$$

where μM= mean and ∑M covariance matrix (provides the covariance between each pair of elements of a random vector in the form of a square matrix) for the *M*-th element. In addition, wk= weight for cluster *M* [69]. The algorithm is trained on these M clusters. Thus, given a new data point, the algorithm finds its distance from each distribution and, therefore, the probability that that point belongs to each cluster. Therefore, if the probability is minimal for a particular cluster, *that is an indication that the data point is an anomaly*.

The GMM is parameterized by means of the mean vectors, the covariance matrices, and the blend weights of all component densities. These parameters are represented collectively by Equation (4) [90].

(4) $$\lambda =\left\{w_{i},\mu_{i},\sum_{i}\right\},$$

where i=1,…,M. It is also possible to use the linear combination of the Gaussian diagonal covariance base to model the correlations between the vector elements of the features. The effect of using a full Gaussian covariance matrix *M* set can also be obtained by using a larger set of diagonal Gaussian covariance.

Now, the *Maximum Likelihood (ML) Parameter Estimation*, used to estimate the parameters of the GMM, is described. In a sequence of *T* training vectors $X=\left\{x_{1},\ldots ,x_{T}\right\}$, the GMM likelihood can be written as Equation (5).

(5) $$p\left(X|\lambda \right)=\prod_{t=1}^{T}p\left(x_{t}|\lambda \right).$$

Given the first model λ, the aim of this method is, to calculate another model λ, based on $p\left(X|\hat{\lambda }\right)\geq p\left(X|\lambda \right)$. Given the above, to estimate the MAP Parameter Estimation is used to calculate GMM parameters via the EM algorithm, which is identical to the *Expectation step of the EM algorithm*. After that, it is combined with a sufficient amount of *old* statistics of the above mixing parameters using a data dependent mixing ratio. Now that the GMM has been described, we move onto describing the electronic design, as well as the implementation of the model in the generated data.

Subsequently, data processing and its modeling are described.

## 4. Data Pre-Processing

A sample of 40,000 points of data was obtained with a frequency of 6 points of data per minute, where each of the components of the bracelet gave the corresponding information through wireless communication. Depending on the values of body temperature, heart rate, and battery of the system, labels were created to simulate the actions or events in different situations, mainly for industrial or transport areas; 4 different classes were generated by the mentioned situations, which are:

- Worker with stable vital signs and non-hostile environment, which we will define as SVSNHE.
- Worker with smooth variation in vital signs, SVVS.
- Worker with vital signs in danger and non-hostile environment, defined here as SVDNHE.
- Worker in danger due to hostile environment, DHE.

For the dataset acquired, the information was taken from workers in the industrial area where the total number of subjects was 7 persons all male with an average age of 34 years and average weight of 72 kg. Each subject was monitored over the course of 15 working days, collecting the aforementioned data, and each user had to fill out a questionnaire at the end of each day to find out how they felt physically, not forgetting any injuries that may have occurred during that week. Since the atypical data were not recurrent during this time, 2 of the 7 workers were asked to simulate falls and situations where their heart rate was abnormal. The environment in which the simulations were carried out was a company in charge of the construction of platforms and infrastructure.

Then, an ANOVA-Fisher analysis was performed, which allowed us to identify that there is a significant variation over the data populations, and, as it can be seen in Table 4, there is no class that shares similar characteristics with another; therefore, it is possible to create clusters over the data.

Given that there is a significant variation in classes, it is necessary to employ methodologies that cluster data and classify actions or environments. Among these methodologies are unsupervised algorithms with classical or fuzzy techniques, such as FCM. In the present work, we focus on grouping by comparing the K-Means method and the GMM.

## 5. Detecting Anomalies with an Electronic Bracelet

**Figure 16:**
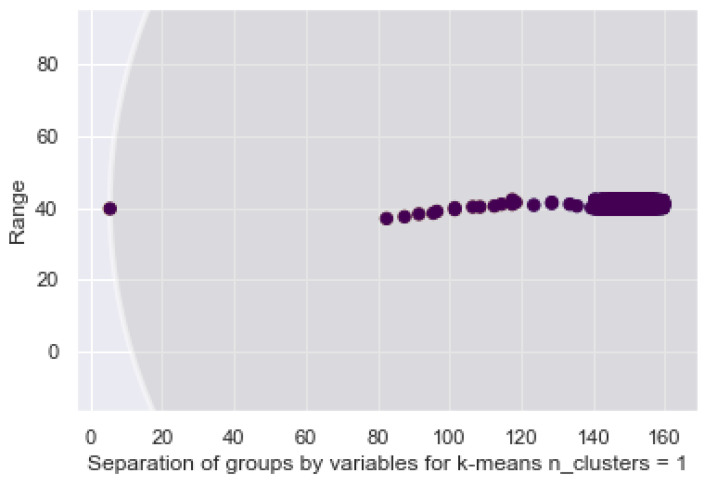
The K-Means between temperature and heartbeat, without constrain.

In order to explore how the bracelet data allows us to find information that may be relevant in different environments, be it work, transportation, health, we modeled the samples with 1 grouping methods. The K-Means looks for similarities and allows us to group them. See Figure 16, although 4 characteristics have been established through Fisher’s analysis, the dispersion of the data without restriction is shown. At first sight, we can already see the way in which information is dispersed.

**Figure 17:**
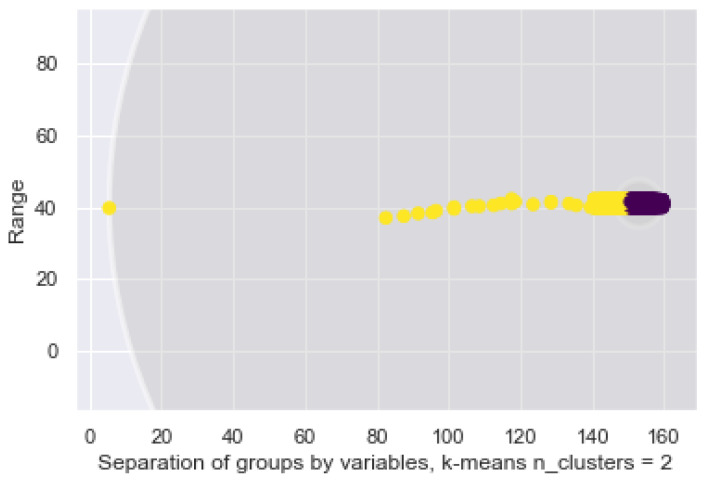
The K-Means between temperature and heartbeat with 2 clusters.

In Figure 17, we place a circle over each of the clusters to delimit the samples for 2 variables that belong to each label. Considering the fact that it is unsupervised learning, it is possible to identify the points that, in spite of their belonging to a specific group, are not grouped where most data are. This makes the problem binary and facilitates the application of a probabilistic approach. Imagine a situation where we only want to determine whether workers in a mine are within an *acceptable* range or are at risk.

**Figure 18:**
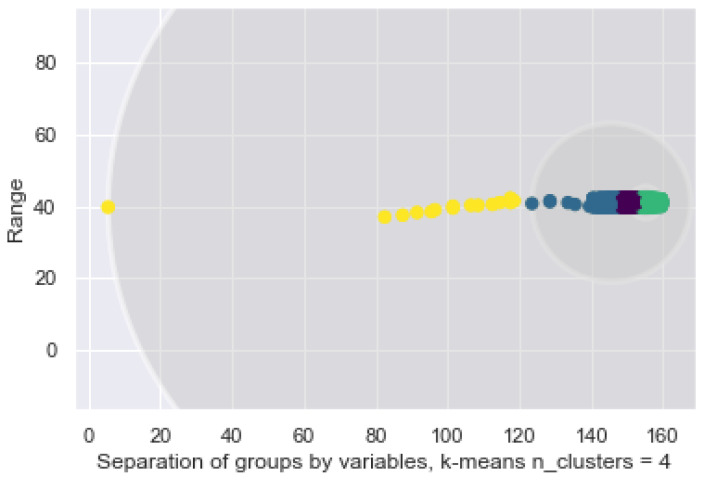
Delimited groupings for 4 labels and 2 variables.

This study shows us that K-Means encounters difficulties when modeling the data and tries to separate them independently, shown previously in Figure 17 and Figure 18 below, as the circles defined by the algorithm overlap. This is because K-Means tries to group the data within the defined radius. However, there should be enough flexibility to find the groupings that do not interfere with others. For example, the yellow samples completely cover the other 3 clusters, although it is shown that each one is defined by a smaller radius, which is the term of the next cluster.

**Figure 19:**
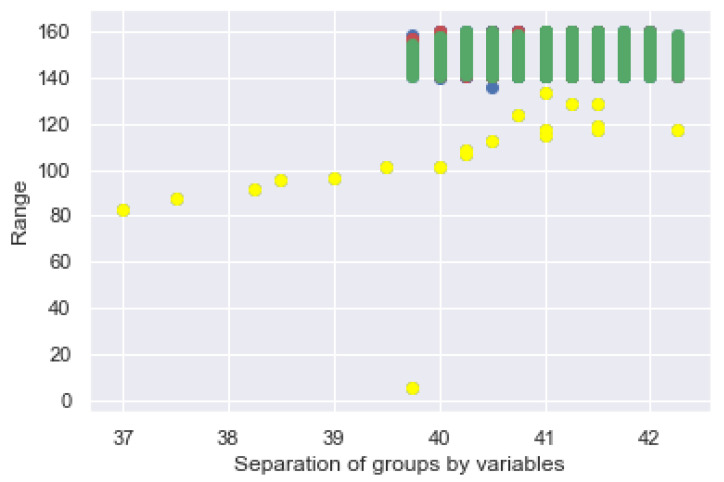
GMM distribution of the 2 classes on the bracelet.

Regarding the following model, the GMM allows us to find the data that fits a distribution. This is an advantage to the bracelet since it enables it to identify the data that are in a range of normality against to those that are not. Abnormal behaviors are identified by the proposed system as possible risks in the user’s environment. Specifically, as seen above, k-means operates in a non-probabilistic manner. It uses simple distance from the center of the cluster to assign membership, which results in poor performance in many real world situations. See Figure 19, where the two biggest data clusters, green and yellow, and the distribution of samples are all grouped in a range of 40 to 42 and 140–160 for the ordinate axis.

**Figure 20:**
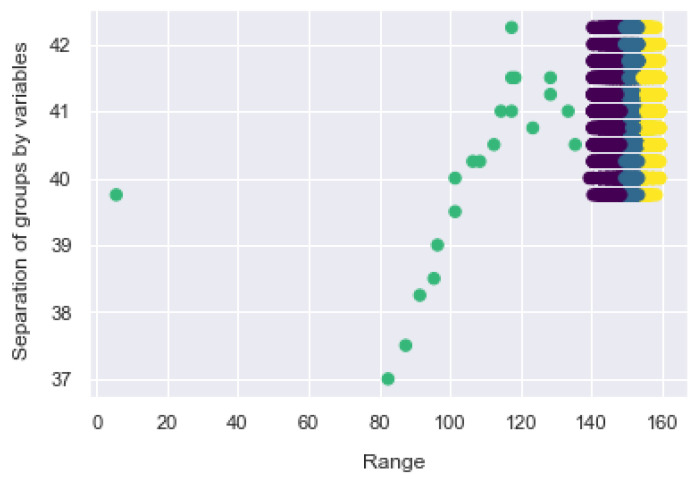
GMM distribution of the 4 classes on the bracelet.

The way of working of the GMM is to find sets in multidimensional data, often called mixtures, to identify the data that are considered normal in a dataset, as shown in Figure 20, where the 4 classes are plotted with respect to the GMM and its groupings. It is possible to see the dispersion of the clusters and the overlap between them.

As shown in Figure 20, it is possible to establish the sample size in proportion to its prediction, where the farthest sample represents the end of the clustering as was the case with K-Means. The GMM uses expectation maximization that chooses initial assumptions for location and shape. As with the K-Means expectation optimization approach, this algorithm can sometimes overlook the overall optimal solution, so it is used multiple random initialization.

**Figure 21:**
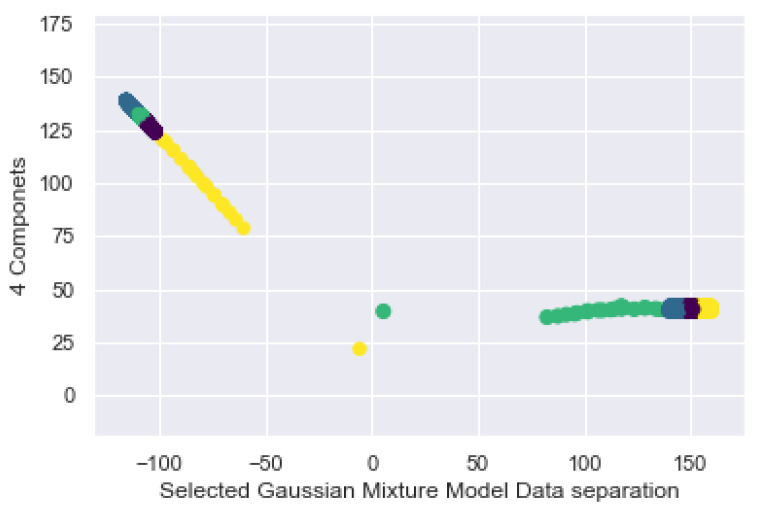
Data separation by GMM.

Figure 21 shows the result; each cluster is associated with a smooth Gaussian model, and not with a hard edge sphere. As in the K-Means expectation optimization approach, this algorithm can sometimes overlook the overall optimal solution, so multiple random initialization is used.

When an anomaly is detected, the information is transmitted via Internet to indicate that there is a possible risk due to the worker’s pulse or temperature. These two clinical variables are extremely important, and they are indispensable in the current situation caused by COVID-19, where an anomalous biological behavior can be detected in order to take action on the matter.

**Figure 22:**
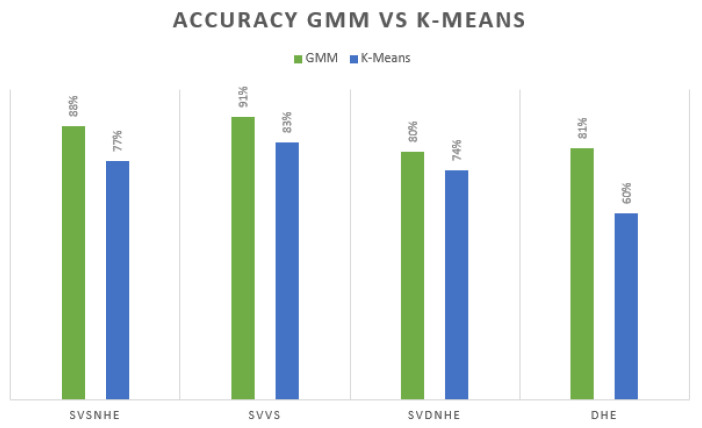
Comparation GMM versus K-Means.

For the training of the system, it is useful to know that we had an unbalanced dataset, where the 80–20 ratio was used for testing and training. Subsequently, real-time tests were performed, where the results are also included. See Figure 22, where we include the comparison of K-Means versus GMM, as it is well known that we can have a better approximation through the GMM.

**Figure 23:**
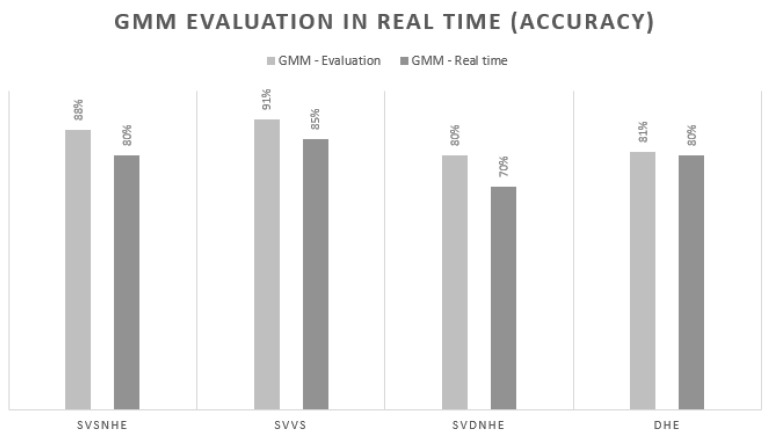
Results of GMM in real time.

However, when we performed the real-time test, we had a decrease in the percentage of accuracy, as shown in Figure 23, and the test was performed over 3 days on 4 different subjects from the same company. A solution for this decrease in percentage may well be to increase the number of subjects from which data was extracted or to increase the sampling time. The decrease in the percentage may be due to other factors, one of them the overlapping of data from one class to another; therefore, one of the proposals for the future is spectral clustering by decomposing the data in its eigenvector representation to maximize the effectiveness of this process. The relevance of our work is in merging emerging technologies that are becoming indispensable in smart cities for smart work with smart health services.

**Figure 24:**
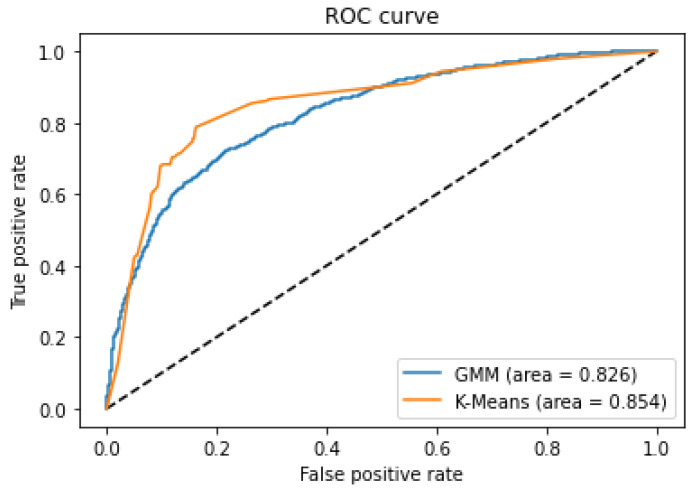
ROC curve for GMM and K-Means, showing the rate of false alarms versus true alarms.

**Figure 25:**
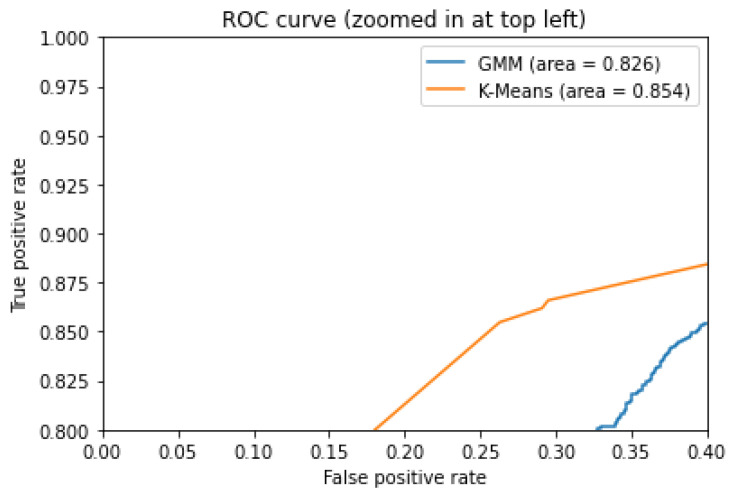
ROC curve for GMM and K-Means, zoomed in at top left.

In addition, included in Figure 24 and Figure 25 is the ROC curve for the two models used specifically for vital signs and the state of the environment, and it can be seen that GMM works better on the number of negative alarms; this comparison helps us to quantitatively see the best option for sending information to the platform.

To send the information to the platform is done in a simple way: a class was made which through the transmission protocol Lab Streaming Layer, where each model receives in real time the inputs with which each model works; once this is known to be mapped from f(x), our hypothesis, or modeled to R, then, again, to the Lab Streaming Layer, the real value is sent along with an identified, which is 0 for GMM and 1 for LSTM. This, in turn, enters the platform that was presented in Figure 6 and Figure 8.

## 6. Conclusions and Discussion

The improvements presented in this work involved the incorporation of new materials and functions in the previously developed protective equipment and an improved centralized platform, offering assistance and visualization services. In conjunction with the bracelet, the platform is highly valuable for risk prevention and medical-health anticipation. The AIoT technological innovations introduced by the bracelet make it possible to monitor aspects that affect the workers’ health. It provides information on the physical parameters and human activity recognition and also analyzes the environment using the capacity provided by Smart Data to detect situations that could lead to occupational illness.

In comparison to state-of-the-art literature, we find that models trained on data from an industrial environment are indeed challenging. In 2016, Reference [56] presented a paper for human activity detection where the authors obtained 92.1% accuracy with a public dataset; however, in our best class classification, we obtained 92% in non-daily tasks, and our activities included moving objects around, carrying and manipulating tools, etc. Therefore, similar accuracy is synonymous with good information acquisition and processing system.

Likewise, an excellent work focused on time series problems, specifically with LSTM and wearable products, was presented in Reference [57], where the authors proposed a framework that was validated across two datasets and convolutional network architectures (specifically, 4 convolutional layers), and its performance was 0.958. In our case, joining all the data on the LSTM gave us a performance that did not exceed 0.70, which is why it was decided to divide the approach of the work into two models; a future work is to implement not a parallel model but to join the advantages of GMM and LSTM in one.

It can also be said that works similar to ours have tried to use different types of regularizations for the deep models [91], where the influence of such improvements was shown. Comparing our work with this one, we can say that our approach is more on the side of using later optimization through Auto Machine Learning, where Gaussian processes and Bayesian optimization will be used to identify the optimal hyperparameter space in the model.

After considering all of the above, it can be concluded that it is extremely important to continue increasing the robustness of devices through information and communication technologies, as well as artificial and electronic intelligence. These technologies enable devices to identify when there is an anomaly in the data obtained by different sensors. Moreover, to transmit the information for decision-making, the present work proposes the detection of anomalies through a multisensorial intelligent bracelet. This bracelet will be implemented in a real environment at a later stage.

The results presented in this paper show that deep learning-based approaches for activity recognition, in the GMM, regularly have associations of the data in each group, and we can also identify the separation of the samples that fall in a range of normality, as in the K-Means optimization approach. Unlike K-Means, the clusters are not identified by a defined diameter, instead, the groups is adapted to a distribution, which makes it is possible to reduce the number of false positives and to identify real risk situations for bracelet wearers. The GMM allows us to identify data that indicate a hazard, even if the groups overlap, this is because it identifies the patterns that define each of the labels.

In the present work, four labels were used to represent different situations. The GMM allowed us to separate them and label those that do not fall within a normal distribution in a multivariate manner. The anomalies are reported to the competent authority by sending the information over the internet. This proposal is applicable to different industries, such as health, where the platform indicates unidentified traits. Moreover, thanks to its heart rate sensor, it can be used by workers in the construction area in order to avoid possible accidents caused by cardiac arrest or fainting due to high body temperatures.

In addition, the research proposes the inclusion of more sensors that work together, as well as a panic button. The system’s adaptive learning enables it to categorize data independently through real-time monitoring. Unlike other state-of-the-art proposals in the literature and already registered patents, we do not establish the classification of labels, we focus on finding critical values in the samples to increase the percentage of true positives and false positives. Part of the future work also involves further exploring the robustness of the proposal with larger datasets.

The BeSafe 2.0 platform and the AIoT bracelet offer a variety of services that meet the needs of different target audiences. Thus, it is necessary to develop a dissemination and communication plan that would be capable of transmitting the generated knowledge and results for their fair and effective use. The dissemination and communication plan must stress the high benefits offered by the proposal, with a high Return on Investment (ROI), given the reduced number of sick leaves and accidents associated with Occupational Risk Prevention (ORP). The market for this type of solutions has been growing in the recent years, showing encouraging results, with an expected growth rate of 15.43% for the period 2017–2021. Moreover, it is expected that, in 2022, this sector will reach a 6% growth, amounting to more than 58 B$.

Future lines of research will focus on identifying variations in audio signals through a microphone and on the use of algorithms, such as LSTM, to identify anomalies in the data or triggers to send information to the point concerned. We will also proposed to use the bracelet in the mobility and logistics sectors, where it could indicate irregularities in traffic anomalies, minimize accidents and even reduce the time taken to transport goods. All these applications would be on the benefit of the economic sectors employing the solution; therefore, they will be explored in a future research.

Finally, it is necessary to continue developing new proposals and protective equipment for different areas of application, adapting the technology to meet their security needs. Among these areas, we are especially interested in providing solutions to people with dementia or patients of some type of neurological disease, such as Alzheimer’s. Thanks to the device, we could prevent people from getting lost or disoriented. Another field that could be covered is the application of the device by people who have suffered some kind of abuse or harassment, as the remote monitoring of the patients’ health would be needed. 

## Figures and Tables

**Table 1:** Proposals related to wearable monitoring and sensor networks in a wrist band.

Bibliography	Sensors Included	Advantages and Disadvantages	Novelty of the Proposal
Shin, D. M. et al. (2013).	GPS, ambient light sensor, accelerometer, wireless communications	It stands out for including GPS and implementing location-based positioning.	It is an intelligent surveillance system, for the use and improvement of living conditions of patients with dementia.
Perez, M. N. et al. (2015).	Optical heart rate sensor, accelerometer, a capacitive sensor, and thermistor	It measures activities, such as exercise and sleep quality, that have not been considered in our work.	Monitor the user’s daily activities, including exercise, sleep quality, heartbeat and food types
Shin, D. et al. (2014).	GPS, ambient light sensor, accelerometer, wireless communications	The work is focused on dementia and includes positioning. It also highlights the algorithm for fall detection.	The main purpose of the bracelet is to prevent dementia patients from getting lost and to detect falls.
Sendra, S. et al. (2018).	Accelerometer, microphone, heart rate, and blood oxygen sensor with photoplethysmography, GPS, elastic band to measure breathing and thermometer	It is focused on disease measurement and incorporates sensors not considered in our article.	Control of chronic diseases of children with remote monitoring constantly with the help of remote devices.
Chen, M. et al. (2018).	Electrocardiogram, temperature and the amount of oxygen in the blood	It measures oxygen in the blood, a sensor that has not been considered in our work.	Monitoring the psychological state of the user using Smart Personal Health Advisor (SPHA) systems
Kajornkasirat, S. et al. (2018).	Heart rate sensor, vibrator, audio support, connection via Bluetooth	Aspects of daily life are detected but no accidents or anomalies.	Counts the steps, the calories burned, monitors our sleep, and analyzes the calories we eat at lunch
Maglogiannis, I. et al. (2014).	Accelerometer, gyroscope and contact sensors, vibrator, magnetometer, ambient light sensor and Bluetooth 4.0	Falls are detected with the CUSUM algorithm and we detect the falls through the IMU itself.	Initial evaluation of fall detection using the CUSUM algorithm
Alsulami, M. H. et al. (2016).	Heart rate sensor	Heart rate is monitored with an expert system called KBS. In our case we extend this to other variables.	The use of smart watches to monitor heart rate in older people using expert system called KBS capable of making decisions and taking action.
Karakaya, M. et al. (2017).	Accelerometer and gyroscope	It only uses an IMU with a KNN classifier as a sensor, so our proposal is more complete	Remote monitoring of elderly people’s activities using Smart Watch using a KNN classifier
Reeder, B. et al. (2016).	Gyroscope, microphones, optical heart rate sensor, contact sensor for temperature measurement and light sensor for sun exposure	Very comprehensive review in which several sensors not covered in our work are used.	Systematic review of the uses of intelligent surveillance for health and well-being with different watch models
Nguyen, D. N. et al. (2017).	Shock sensor, microphone, pulse sensor, temperature sensor and GPS	Detection of parameters for anomaly detection using a microphone as an extra to our work.	Smart Watch with automatic voice recording and alarm
Parara, A., & Sekka, S. (2016).	Heart rate sensor, GPS, touch screen and microphone	A system including positioning and a microphone as additional components is presented.	Intelligent user care security surveillance device
Mukhopadhyay, S. C. (2014).	Body temperature sensor, heart rate meter with photoplethysmography (PPG), microphone, camera, accelerometer, and electrocardiogram	It focuses on Human Behavior Activity and is not aimed at alarm detection.	Review of wearable sensors for monitoring human activity
Gope, C. (2015).	Accelerometer, GPS, panic button	It is aimed at the detection of epileptic movements and does not cover other areas of interest in our case.	Smart Watch for surveillance and monitoring of seizures / abnormal movement activities or epileptic seizures.
Wile, D. J. et al. (2014).	Accelerometer	It is aimed at tremor analysis and does not cover other areas of interest in our case.	Smart Watch accelerometry for tremor analysis and diagnosis
Kumari, P. et al. (2017)	Electroencephalogram (EEG), electrooculogram (EOG), electromyography (EMG), electrocardiogram (ECG)	This is similar work that focuses on aspects of monitoring people rather than detecting alarms caused by accidents.	Review of wearables and multimodal interface for human activity monitoring
Manisha, M. et al. (2016)	Heart rate and blood pressure sensor	The application is heart attack detection. So it only uses sensors aimed at detecting heart attacks.	Device targeting heart disease, monitoring heartbeats and blood pressure, to try to reduce the number of deaths due to heart attacks
Dhull, R. et al. (2020)	Failure of respiratory system of human, body temperature, heart rate, and blood pressure.	The smartwatch is used for COVID detection and the sensors it implements and its software are closed to this application.	Discuss the design, principle of operation and features of different smartwatches
Adjiski, V. et al. (2019)	Accelerometer, gyroscope, magnometer, and heart rate sensor	It is dedicated to mining and the smartwatch does not implement fall detection.	Real-time safety situation awareness and predict health and safety incidents before they occur

**Table 2:** Identification of common risk situations in the worker’s environment and electronic components.

Risk Factors	Associated Hazards	Solution
Heart rate	- Heart attack and irregular heartbeat	- Wearing a heart rate sensor on the wrist
Temperature	- Extreme temperature changes leading to a heat stroke - Unhealthy temperature for work that may indicate fever or hypothermia	- Implementation of temperature sensors in the bracelet
Operator Movement	- Slips, trips and falls - Blows to the worker’s hand	- The use of IMU capable of detecting falls or impacts - Integration of sensitive force resistors in the bracelet of the operator.
Reporting an accident	- Falls, intoxication, fire, collapse, heart attack, loss of consciousness, among others.	- Resistive touch pad

**Table 3:** Technical specifications of the sensors selected for the device.

Component	Characteristics	Description
Thermocouple Type-K	- Precision: ±1 ºC - Output range: −6 to 20 mV	Glass braid insulated stainless steel tip which can be used in high temperature.
Heart Rate Monitor Sensor	- Input Voltage (Vin): 3.3–6 V (5 V recommended) - Output Voltage: 0–Vin (Analog), 0/Vin (Digital) - Operating current: <10 mA	It is based on PPG techniques, to detect blood volume changing in the microvascular bed of tissues
BMI160 Inertial sensor (IMU)	- Sensitivity (typ.) Acc. ±2 g:16,384 LSB/g, ±4 g:8192 LSB/g, ±8 g:4096 LSB/g, ±16 g:2048 LSB/g - Sensitivity (typ.) Gyro. ±125º/s:262.4 LSB/º/s, ±250º/s:131.2 LSB/º/s, ±500º/s:65.6 LSB/º/s - TCS (typ.) (A): ±0.03%/K (G): ±0.02%/K - Nonlinearity (typ.) (A): 0.5%FS (G): 0.1 %FS - Offset (typ.) (A): ±40 mg (G): ±3º/s - TCO (typ.) (A): ±1.0 mg/K (G): 0.05º/s/K	It is an inertial measurement unit (IMU) consisting of a state-of-art 3 axis, low-g accelerometer, and a low power 3 axis gyroscope.
Square Force-Sensitive Resistor (FSR)	- Actuation Force ∼0.2 N min - Force Sensitivity Range: ∼0.2 N–20 N - Force Repeatability Single Part +/− 2% - Force Repeatability Part to Part +/− 6% (Single Batch)	FSRs are sensors that allow to detect physical pressure, squeezing, and weight.

**Table 4:** Fisher analysis performed to determine the components to be used.

Grouping Information Using the Fisher LSD Method and 95% Confidence						
Temp	N	Mean	Grouping			
36.50	168	5.000	A			
42.00	303	4.0264		B		
41.75	761	4.01840		B		
40.50	1051	4.01808		B		
40.25	809	4.01731		B		
41.00	2183	4.01466		B		
40.75	1601	4.01437		B		
41.25	1520	4.01118		B		
41.50	1131	4.00973		B		
40.00	301	4.0033		B		
42.25	166	3.99398		B		
39.50	1	2.0000			C	
39.00	1	2.0000			C	
38.50	1	2.0000			C	
38.25	1	2.0000			C	
37.50	1	0.0000				D
37.50	1	0.0000				D

## Data Availability

Not applicable.

## Sample Availability

Samples of the compounds are available from the authors.
